# Crystal structure and Hirshfeld surface analysis of mono/bis­(aqua-κ*O*)[*N*-(2-oxido­benzyl­idene)valinato-κ^3^ *O*,*N*,*O*′]copper(II): dimeric Schiff base copper(II) complexes having different numbers of coordinated water mol­ecules

**DOI:** 10.1107/S2056989023002487

**Published:** 2023-03-21

**Authors:** Yukihito Akiyama, Soma Suzuki, Shintaro Suda, Yuji Takiguchi, Daisuke Nakane, Takashiro Akitsu

**Affiliations:** a Department of Chemistry, Faculty of Science, Tokyo University of Science, 1-3 Kagurazaka, Shinjuku-ku, Tokyo 162-8601, Japan; Universidade de Sâo Paulo, Brazil

**Keywords:** Schiff base complex, copper, amino acid, Hirshfeld analysis, crystal structure

## Abstract

The mol­ecular structure of the title compound, [Cu(C_12_H_13_N_2_O_3_)(H_2_O)_2_]·[Cu(C_12_H_13_N_2_O_3_)(H_2_O)], consists of two different mol­ecules in the asymmetric unit. Both of the structures consist of a tridentate ligand synthesized from l-valine and salicyl­aldehyde, and one water mol­ecule or two water mol­ecules coordinating to Cu^II^. They have a square-planar (mol­ecule 1) or a square-pyramidal (mol­ecule 2) coordination geometry. In the crystal, the mol­ecules form intra- and inter­molecular O—H⋯O hydrogen bonds involving the coordinated water mol­ecules and other sites. A Hirshfeld surface analysis indicated that the most important contributions to the packing are from H⋯H [52.9% (mol­ecule 1) and 51.1% (mol­ecule 2)] and H⋯O/ O⋯H [21.2% (mol­ecule 1) and 25.8% (mol­ecule 2)] contacts. In addition, an electrostatic potential map was also obtained from DFT calculations to support the discussion of the inter­molecular inter­actions.

## Abstract 2

The mol­ecular structure of the title compound, [Cu(C_12_H_13_N_2_O_3_)(H_2_O)_2_]·[Cu(C_12_H_13_N_2_O_3_)(H_2_O)], consists of two different mol­ecules in the asymmetric unit. Both of the structures consist of a tridentate ligand synthesized from l-valine and salicyl­aldehyde, and one water mol­ecule or two water mol­ecules coordinating to Cu^II^.

## 1. Chemical context

**Figure 1:**
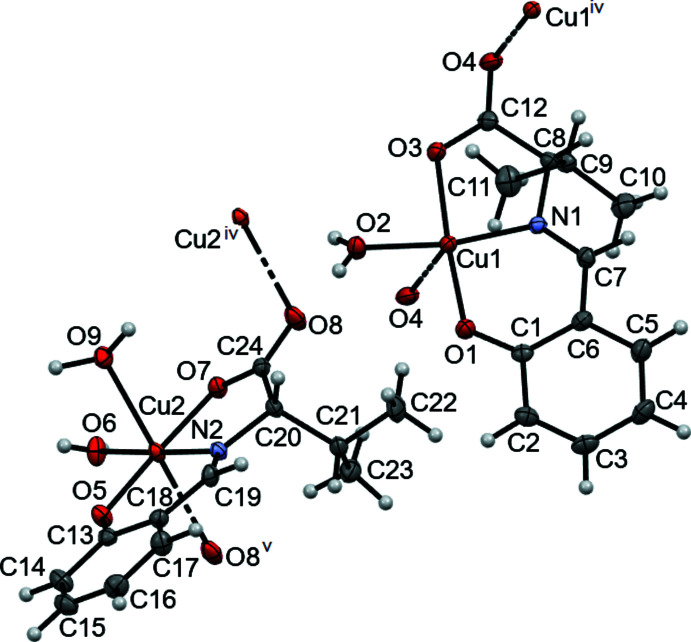
The mol­ecular structure of the title compound. Displacement ellipsoids are drawn at the 50% probability level. [Symmetry codes: (iv) *x* − 1, *y*, *z*; (v) *x* + 1, *y*, *z*.]

Amino acid Schiff bases, which can easily be synthesized by mixing primary amines and carbonyl components, are organic ligands having an azomethine (C=N) group. They play an important and diverse role in coordination chemistry (Qiu *et al.*, 2008 ▸; Li *et al.*, 2010 ▸; Xue *et al.*, 2009 ▸; Katsuumi *et al.*, 2020 ▸). We recently published a review (Akitsu *et al.*, 2022 ▸) of the synthesis of amino acid Schiff base–metal complexes. According to the literature, in general, Schiff bases and their metal complexes are multipurpose compounds and are extensively utilized in many research and industrial applications. These compounds can be utilized alone or for the preparation of various hybrid materials, such as, for example, supra­molecular elastomers with imine-functionalized polysiloxanes (Hu *et al.*, 2010 ▸), conducting metallopolymers for electrochemical sensing (González *et al.*, 2021 ▸), while recently reported co-crystals of a Schiff base with lead iodide perovskite show photo-triggered ferroelectricity (Deng *et al.*, 2022 ▸). Furthermore, Schiff base complexes are considered to be an important class of organic compounds with a wide range of biological properties, including free radical scavenging, anti­bacterial, anti­tumor activities (Mo *et al.*, 2022 ▸). In our laboratory, novel mono-chlorinated Schiff base Cu^II^ complexes have been synthesized and their anti­bacterial activities tested against Gram-positive and Gram-negative bacteria; the most active were then tested for their anti­oxidant activities, and as *E. coli*, in particular, was found to be sensitive to these compounds, their inter­action with this bacterium was investigated (Otani *et al.*, 2022 ▸). Microwave irradiation is suitable for the synthesis of amino acid Schiff bases Cu^II^ complexes in order to shorten the synthesis time and to obtain high purity. In the present study, the title compound was therefore synthesized by using microwave irradiation (Otani *et al.*, 2022 ▸). Differences in chemical properties as a result of differences in structure are remarkable and it is important to report different crystal structures to discuss these features. In this study, we report the structure of the title Schiff base Cu^II^ complex (Fig. 1 ▸) derived from l-valine and salicyl­aldehyde, which has a similar structure to that of one we reported previously (Katsuumi *et al.*, 2020 ▸). 
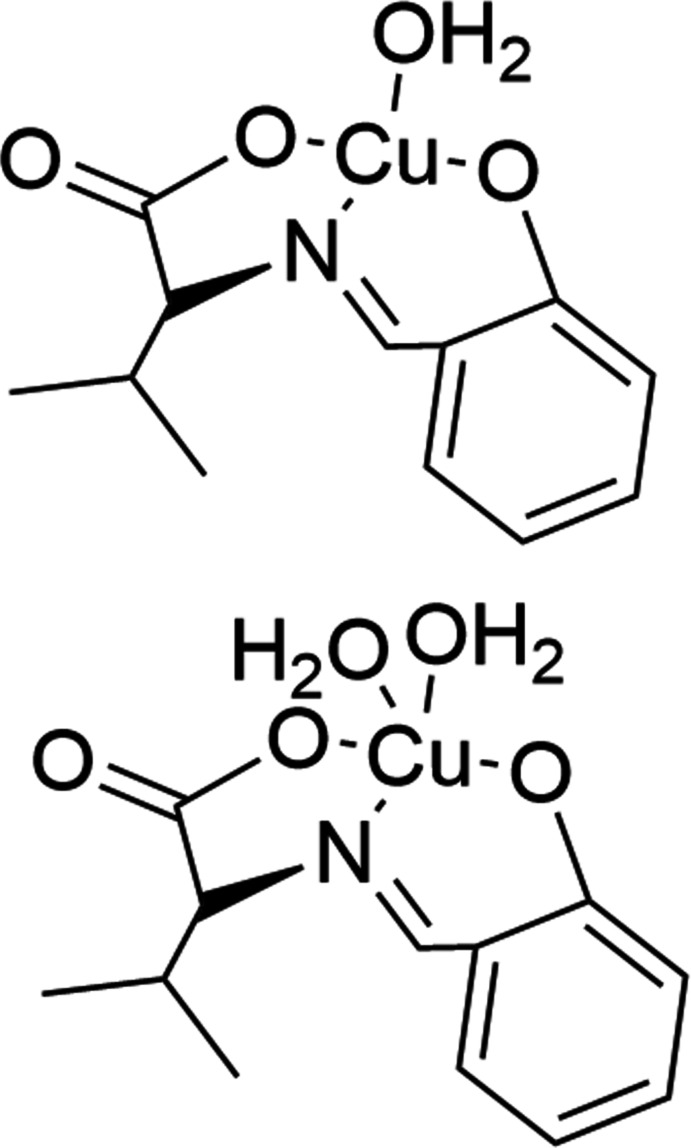


## 2. Structural commentary

The asymmetric unit of the title compound consists of two different mol­ecules, mol­ecule 1 (containing atom Cu1) and mol­ecule 2 (including Cu2). In mol­ecule 1, Cu1 has square-planar geometry, being coordinated by a tridentate ligand synthesized from l-valine and salicyl­aldehyde in the equatorial plane and by one water mol­ecule. The C7=N1 double-bond distance is 1.284 (3) Å, which is close to the typical C=N double bond length for imines (Katsuumi, *et al.*, 2020 ▸). The Cu1—O1, Cu1—O2 and Cu1—O3 bond lengths are 1.8995 (15), 1.9339 (16), and 1.9629 (15) Å, respectively, close to a typical Cu—O coordination bond length (Katsuumi *et al.*, 2020 ▸). The Cu1—N1 bond length of 1.9205 (16) Å corresponds to typical Cu—N bond length (Katsuumi *et al.*, 2020 ▸). The lengths of these four coordination bonds in mol­ecule 1 are almost the same. Finally, the bond the other inter­acting O atom, Cu1⋯O4(1 + *x*, *y*, *z*), is 2.6586 (17) Å.

In contrast, in mol­ecule 2 the Cu2 atom exhibits a square-pyramidal geometry, being coordinated by the same tridentate ligand in the equatorial plane and by two water mol­ecules in the equatorial and axial positions. The C19=N2 double-bond distance is 1.278 (3) Å, which is again close to the typical C=N double-bond length for imines (Katsuumi *et al.*, 2020 ▸). The Cu2—O5, Cu2—O6, and Cu2—O7 bond lengths are 1.9432 (14), 1.9411 (15), and 1.9956 (14) Å, respectively, which are close to a typical Cu—O bond length (Katsuumi *et al.*, 2020 ▸). The Cu2—N2 bond length of 1.9243 (17) Å corresponds to a typical Cu—N bond length (Katsuumi *et al.*, 2020 ▸). Again, these four coordination bonds are almost the same length. The bond lengths involving the other inter­acting O atoms are Cu2⋯O8(1 + *x*, *y*, *z*) = 2.7937 (16) Å and Cu2—O9 = 2.3663 (16) Å; the latter is longer than Cu2—O6 because of the pseudo-Jahn–Teller effect. The Cu2—O6 bond is slightly longer than Cu1—O2 as a result of the crowding that occurs as the number of coordinating water mol­ecules increases.

## 3. Supra­molecular features

**Figure 2:**
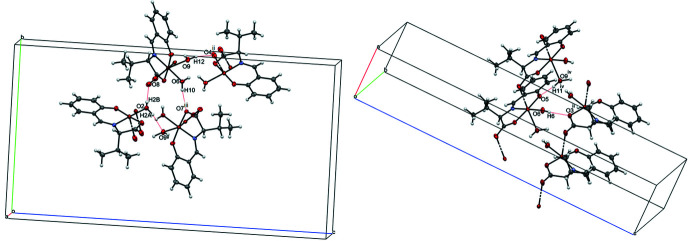
A view of the intra- and inter­molecular O—H⋯O hydrogen bonds, shown as dashed lines. [Symmetry codes: (ii) *x* + 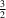 , −*y* + 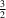 , −*z* + 1; (iii) *x* + 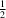 , −*y* + 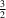 , −*z* + 1; (iv) *x* − 1, *y*, *z*; (v) *x* + 1, *y*, *z*.]

Six inter­molecular O—H⋯O hydrogen bonds (Table 1 ▸ and Fig. 2 ▸) are observed in the unit cell; (O2—H2*B*⋯O8, O2—H2*A*⋯O9^i^, O6—H6⋯O3^ii^, O6—H10⋯O7^iii^, O9—H11⋯O5^iv^, and O9—H12⋯O4^ii^; symmetry codes as in Table 1 ▸). The angle O4(1 + *x*, *y*, *z*)—Cu1—N1 [102.50 (6)^°^] is tilted by much more than 90° as a result of the O9—H12⋯O4^ii^ hydrogen bond. Similarly, the angle O8(1 + *x*, *y*, *z*)—Cu2—N2 [98.58 (6)°] is tilted by more than 90° because of the effect of the O2—H2*B*⋯O8 hydrogen bond. In the crystal, the mol­ecules form an infinite chain as a result of the inter­action of these six hydrogen bonds and the Cu^II^ atom with the carbonyl groups of the ligands (Figs. 2 ▸ and 3 ▸). These six hydrogen bonds also form strong inter­actions between mol­ecules 1 and 2 unit. The equatorial planes of ligands in the same type of mol­ecule are parallel to each other, while those of mol­ecules 1 and mol­ecule 2 inter­sect at an angle of 52.29 (2)°.

**Figure 4:**
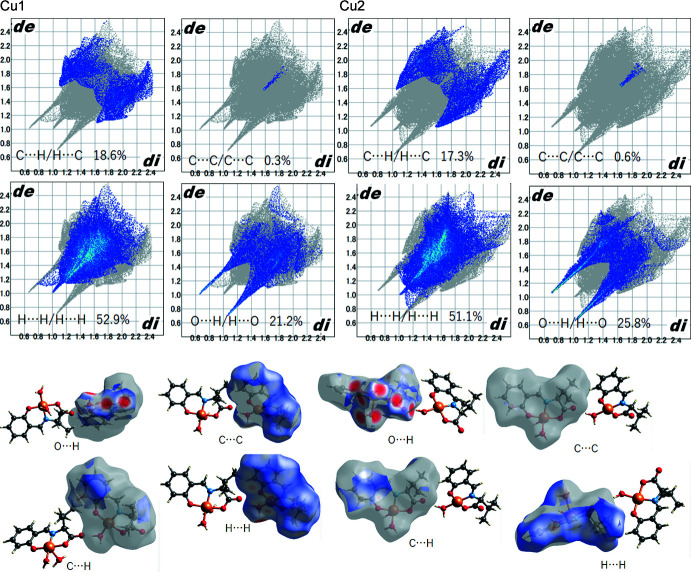
Hirshfeld surfaces mapped over *d*_norm_ and two-dimensional fingerprint plots.

Hirshfeld surface analysis (Spackman & Jayatilaka, 2009 ▸; McKinnon *et al.*, 2007 ▸) was performed to better understand the inter­molecular inter­actions and contacts. The inter­molecular O—H⋯O hydrogen bonds are indicated by bright-red spots appearing near atoms O4, O5, O8, O9 and water H atoms on the Hirshfeld surfaces mapped over *d*_norm_ and by two sharp spikes of almost the same length in the region 1.6 < (*d*_e_ + *d*_i_) < 2.0 Å in the 2D fingerprint plots (Fig. 4 ▸). In mol­ecule 1, the contributions to the packing from H⋯H, C⋯C, C⋯H/H⋯C and H⋯O/O⋯H contacts are 52.9, 0.3, 18.6 and 21.2%, respectively, and in mol­ecule 2, the contributions of the H⋯H, C⋯C, C⋯H/H⋯C and H⋯O/O⋯H contacts are 51.1, 0.6, 17.3 and 25.8%, respectively (Fig. 4 ▸). A common feature of the two structures is the high values for the contributions of H⋯H/H⋯H and C⋯H/H⋯C contacts, with H⋯H/H⋯H representing the influence of van der Waals forces and C⋯H/H⋯C representing the influence of C—H⋯π inter­actions as a result of the presence of aromatic rings in the structures. The reason for the low C⋯C/C⋯C ratio is thought to be that the aromatic rings do not overlap, as indicated by the packing structure, and thereby the contribution of π–π stacking is low. Compared to mol­ecule 1, mol­ecule 2 has a larger number of water mol­ecules and a higher H⋯O/O⋯H value, which seems to have resulted in a larger contribution from hydrogen bonding and corresponding decreases in the C⋯H/H⋯C and H⋯H/H⋯H values.

## 4. DFT calculations

Quantum chemical calculations were carried out to compare the structure in the gas phase with that of in the crystal. The optimized structure of the title compound in the gas phase was calculated by density functional theory (DFT) and the calculation was performed using the *Gaussian 09W* software package (Revision D.02; Frisch *et al.*, 2009 ▸). The Lanl2DZ basis set was applied to the central metal atom (Cu), the 6-31G(d) basis set to the other atoms (C, O, N, H), and the effective core potential (ECP) to the central metal. Calculations were performed for square-pyramidal and square-planar geometries. The initial structure was obtained from X-ray refinement data. However, in the optimizing calculations for the square-pyramidal geometry, the mol­ecule was unable to maintain the square-pyramidal structure, and the axial water mol­ecules moved outside the first coordination sphere of the central metal atom. This indicates that the square-pyramidal structure of this mol­ecule is not stable in the gas phase. The axial water mol­ecules are stabilized by hydrogen bonds with neighboring mol­ecules and the square-pyramidal structure is considered to be preserved.

**Figure 5:**
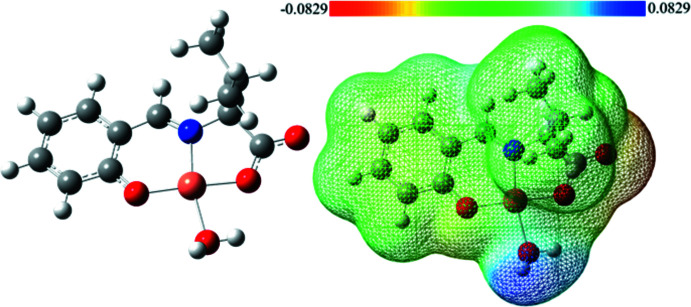
Optimized structure and electrostatic potential map for the title compound.

The bond lengths and bond angles of the square-planar sites are generally consistent between the crystal structure and the optimized structure (Table 2 ▸). In the DFT-optimized structure, the hydrogen atoms of the water mol­ecule are oriented towards the carboxyl group and appear to be involved in an inter­molecular hydrogen bond. The orientation of the water mol­ecules in the crystal is largely influenced by the hydrogen-bonding network. It can be seen that the carb­oxy group of the ligand is charged electron rich, while the hydrogen atom of water shows an electron deficiency. Therefore, the carb­oxy group is considered to be an electron donor and the water hydrogen atoms are electron acceptor, which is also consistent with the crystal structure (Fig. 5 ▸).

## 5. Database survey

A search in the Cambridge Structural Database (CSD, Version 5.43, update of March 2022; Groom *et al.*, 2016) for similar structures returned three relevant entries: (aqua-*O*)(methanol-*O*)[*N*-salieyl­idene-l-threoninato]copper(II) (YUYFUW; Katsuumi *et al.*, 2020 ▸), (aqua-*O*)[*N*-salieyl­idene-l-threonin­ato]copper(II) (SLCDCU; Korhonen & Hämäläinen, 1981 ▸), and (aqua-*O*)[*N*-salieyl­idene-l-valinato]copper(II) (SAV­ACU; Korhonen & Hamalainen, 1979 ▸). In the crystal of YUYFUW, a chain along the *a*-axis direction is formed by one hydrogen bond while the other two hydrogen bonds form a hydrogen-bonded ring. The mol­ecules are packed in a double-column along the *a*-axis direction *via* these three hydrogen bonds. In the crystal of SLCDCU, two mol­ecules form square planes by two inter­molecular hydrogen bonds. The crystal of SAVACU has a very similar structure to that of the title complex. In SAVACU, there are two mol­ecules in the unit cell. They have a coordination water per mol­ecule in the unit cell, while the title compound has one or two coordination waters per mol­ecule in the unit cell.

## 6. Synthesis and crystallization

l-valine (117.7 mg, 1.01 mmol) was reacted with salicyl­aldehyde (121.5 mg, 0.996 mmol) in methanol (20 mL), which was treated with microwave irradiation at 358 K for 5 min to yield a yellow ligand solution. Copper(II) acetate monohydrate (200.9 mg, 1.01 mmol) was added to the ligand solution and treated with microwave irradiation at 358 K for 5 min to yield a green solution. For recrystallization, the green solution was placed in the air at 300 K for several days, and the title complex was obtained (135.4 mg, 0.437 mmol, yield 43.9%) as green needle-shaped single crystals suitable for single-crystal X-ray diffraction experiments. Elementary analysis: found: C, 47.78; H, 5.12; N, 4.61%. Calculated: C_24_H_32_Cu_2_N_2_O_9_, C, 46.52; H, 5.21; N, 4.52%. IR (KBr): 1026 cm^−1^ (*m*), 1076 cm^−1^ (*w*), 1134 cm^−1^ (*w*), 1152 cm^−1^ (*m*), 1197 cm^−1^ (*m*), 1286 cm^−1^ (*w*), 1317 cm^−1^ (*m*), 1370 cm^−1^ (*m*), 1450 cm^−1^ (*s*, C=C double bond), 1455 cm^−1^ (*m*), 1535 cm^−1^ (*m*), 1600 cm^−1^ (*s*, C=O double bond), 1639 cm^−1^ (*s*, C=N double bond), 2960 cm^−1^ (*w*), 3300 cm^−1^ (*br*, O—H). UV–vis: 269 nm (ɛ = 25000 L mol^−1^ cm^−1^, *n*–π^*^); 367 nm (ɛ = 9330 L mol^−1^ cm^−1^, π–π^*^); 664 nm (ɛ =163 L mol^−1^ cm^−1^, *d*–*d*).

## 7. Refinement

Crystal data, data collection and structure refinement details are summarized in Table 3 ▸. All C-bound H atoms were placed in geometrically calculated positions (C—H = 0.93–0.98 Å) and were constrained using a riding model with *U*_iso_(H) = 1.2*U*_eq_(C) for *R*2CH and *R*3CH H atoms and 1.5*U*_eq_(C) for the methyl H atoms. The O-bound H atoms were located based on a difference-Fourier map and refined isotropically (H2*B*, H10, H11, and H12) or using riding model (H2*A* and H6) with O—H = 0.82 Å. Water H atoms were freely refined.

## Supplementary Material

Crystal structure: contains datablock(s) global, I. DOI: 10.1107/S2056989023002487/ex2068sup1.cif

Structure factors: contains datablock(s) I. DOI: 10.1107/S2056989023002487/ex2068Isup2.hkl

Figure S1. FT-IR spectra of the complex. IR (KBr): 1026 cm-1(m), 1076 cm-1(w), 1134 cm-1(w), 1152 cm-1(m), 1197 cm-1(m), 1286 cm-1(w), 1317 cm-1(m), 1370 cm-1(m), 1450 cm-1(s, C C double bond), 1455 cm-1(m), 1535 cm-1(m), 1600 cm-1(s, C O double bond), 1639 cm-1(s, C N double bond), 2960 cm-1(w), 3300 cm-1(br, OH). DOI: 10.1107/S2056989023002487/ex2068sup3.tif

CCDC reference: 2248369

Additional supporting information:  crystallographic information; 3D view; checkCIF report

## Figures and Tables

**Figure 3:**
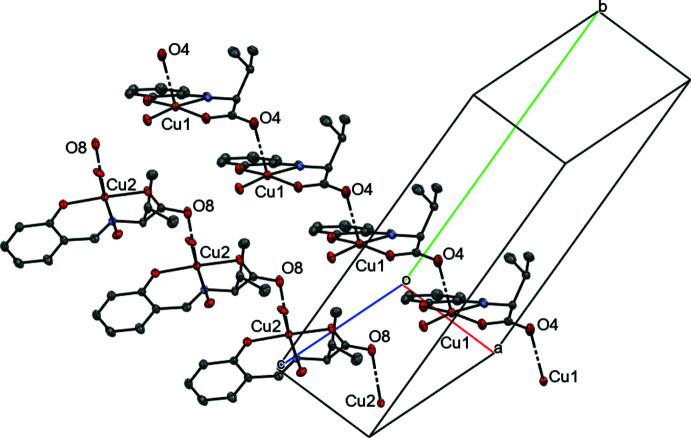
The chains resulting from the coordination bonding of the carbonyl groups to the copper(II) atoms. Hydrogen atoms are omitted for clarity.

**Table 1:** Hydrogen-bond geometry (Å, °)

*D*—H⋯*A*	*D*—H	H⋯*A*	*D*⋯*A*	*D*—H⋯*A*
O2—H2*A*⋯O9^i^	0.83	1.87	2.688 (2)	167
O2—H2*B*⋯O8	0.72 (3)	1.93 (3)	2.645 (2)	169 (4)
O6—H6⋯O3^ii^	0.72	2.07	2.784 (2)	171
O6—H10⋯O7^iii^	0.77 (3)	2.01 (3)	2.764 (2)	169 (3)
O9—H11⋯O5^iv^	0.94 (2)	1.80 (2)	2.738 (2)	172 (3)
O9—H12⋯O4^ii^	1.01 (2)	1.74 (2)	2.703 (2)	157 (3)

Symmetry codes: (i) 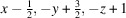 ; (ii) 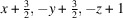 ; (iii) 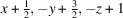 ; (iv) 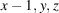 .

**Table 2:** Comparison of selected (X-ray and DFT) bond lengths and angles (Å, °)

Square-planar mol­ecule 1		Square-pyramidal mol­ecule 2		
Bonds/Angles	X-ray	Bonds/Angles	X-ray	B3LYP/6–31G(*d*) (C, N, O, H); Lanl2DZ (Cu)
Cu1—O1	1.8955 (15)	Cu2—O5	1.9432 (14)	1.881
Cu1—O2	1.9339 (16)	Cu2—O6	1.9411 (15)	2.079
Cu1—O3	1.9629 (15)	Cu2—O7	1.9956 (14)	1.913
Cu1—N1	1.9205 (16)	Cu2—N2	1.9243 (17)	1.944
O2⋯O3	2.707 (2)	O6⋯O7	2.903 (2)	2.611
N1—Cu1—O2	170.16 (7)	N2—Cu2—O6	177.73 (7)	167.07

**Table 3:** Experimental details

Crystal data	
Chemical formula	[Cu(C_12_H_13_N_2_O_3_)(H_2_O)_2_]·[Cu(C_12_H_13_N_2_O_3_)(H_2_O)]
*M*_r_	619.59
Crystal system, space group	Orthorhombic, *P*2_1_2_1_2_1_
Temperature (K)	173
*a*, *b*, *c* (Å)	5.2966 (3), 15.8698 (10), 29.5216 (16)
*V* (Å^3^)	2481.5 (2)
*Z*	4
Radiation type	Mo *K*α
μ (mm^−1^)	1.77
Crystal size (mm)	0.30 × 0.30 × 0.20

Data collection	
Diffractometer	Bruker D8 QUEST
Absorption correction	Multi-scan (*SADABS*; Krause *et al.*, 2015 ▸)
*T*_min_, *T*_max_	0.46, 0.72
No. of measured, independent and observed [*I* > 2σ(*I*)] reflections	31718, 6889, 6586
*R*_int_	0.036
(sin θ/λ)_max_ (Å^−1^)	0.732

Refinement	
*R*[*F* ^2^ > 2σ(*F* ^2^)], *wR*(*F* ^2^), *S*	0.024, 0.053, 1.04
No. of reflections	6889
No. of parameters	358
No. of restraints	6
H-atom treatment	H atoms treated by a mixture of independent and constrained refinement
Δρ_max_, Δρ_min_ (e Å^−3^)	0.28, −0.31
Absolute structure	Flack *x* determined using 2499 quotients [(*I* ^+^)−(*I* ^−^)]/[(*I* ^+^)+(*I* ^−^)] (Parsons *et al.*, 2013 ▸)
Absolute structure parameter	0.011 (4)

Computer programs: *SAINT* (Bruker, 2019 ▸), *SHELXT2018/2* (Sheldrick, 2015*a* ▸), *SHELXL2018/3* (Sheldrick, 2015*b* ▸), and *ShelXle* (Hübschle *et al.*, 2011 ▸).

## supplementary crystallographic information

### Crystal data

**Table:**

[Cu(C_12_H_13_N_2_O_3_)(H_2_O)_2_]·[Cu(C_12_H_13_N_2_O_3_)(H_2_O)]	*D*_x_ = 1.658 Mg m^−^^3^
*M**_r_* = 619.59	Mo *K*α radiation, λ = 0.71073 Å
Orthorhombic, *P*2_1_2_1_2_1_	Cell parameters from 9895 reflections
*a* = 5.2966 (3) Å	θ = 2.4–31.1°
*b* = 15.8698 (10) Å	µ = 1.77 mm^−^^1^
*c* = 29.5216 (16) Å	*T* = 173 K
*V* = 2481.5 (2) Å^3^	Prism, green
*Z* = 4	0.30 × 0.30 × 0.20 mm
*F*(000) = 1280	

### Data collection

**Table:**

Bruker D8 QUEST diffractometer	6586 reflections with *I* > 2σ(*I*)
Detector resolution: 7.3910 pixels mm^-1^	*R*_int_ = 0.036
φ and ω scans	θ_max_ = 31.4°, θ_min_ = 2.4°
Absorption correction: multi-scan (SADABS; Krause *et al.*, 2015)	*h* = −7→7
*T*_min_ = 0.46, *T*_max_ = 0.72	*k* = −22→22
31718 measured reflections	*l* = −40→40
6889 independent reflections	

### Refinement

**Table:**

Refinement on *F*^2^	Hydrogen site location: mixed
Least-squares matrix: full	H atoms treated by a mixture of independent and constrained refinement
*R*[*F*^2^ > 2σ(*F*^2^)] = 0.024	*w* = 1/[σ^2^(*F*_o_^2^) + (0.0222*P*)^2^ + 0.2409*P*] where *P* = (*F*_o_^2^ + 2*F*_c_^2^)/3
*wR*(*F*^2^) = 0.053	(Δ/σ)_max_ = 0.005
*S* = 1.04	Δρ_max_ = 0.28 e Å^−^^3^
6889 reflections	Δρ_min_ = −0.31 e Å^−^^3^
358 parameters	Absolute structure: Flack *x* determined using 2499 quotients [(*I*^+^)-(*I*^-^)]/[(*I*^+^)+(*I*^-^)] (Parsons *et al.*, 2013)
6 restraints	Absolute structure parameter: 0.011 (4)

### Special details

**Table:**

Geometry. All esds (except the esd in the dihedral angle between two l.s. planes) are estimated using the full covariance matrix. The cell esds are taken into account individually in the estimation of esds in distances, angles and torsion angles; correlations between esds in cell parameters are only used when they are defined by crystal symmetry. An approximate (isotropic) treatment of cell esds is used for estimating esds involving l.s. planes.

### Fractional atomic coordinates and isotropic or equivalent isotropic displacement parameters (Å^2^)

**Table:**

	*x*	*y*	*z*	*U*_iso_*/*U*_eq_	
Cu1	0.11262 (5)	0.88425 (2)	0.63867 (2)	0.01477 (6)	
O1	0.3595 (3)	0.83347 (9)	0.67629 (5)	0.0192 (3)	
N1	−0.0502 (3)	0.94302 (10)	0.68746 (6)	0.0143 (3)	
C1	0.3607 (4)	0.83347 (12)	0.72030 (7)	0.0161 (4)	
O2	0.2196 (3)	0.82454 (11)	0.58489 (5)	0.0196 (3)	
H2A	0.244 (5)	0.8586 (12)	0.5641 (8)	0.029*	
H2B	0.302 (7)	0.788 (2)	0.5828 (10)	0.037 (9)*	
Cu2	1.10130 (5)	0.59080 (2)	0.52618 (2)	0.01400 (6)	
N2	0.9488 (3)	0.52729 (10)	0.57450 (6)	0.0142 (3)	
C2	0.5531 (4)	0.78889 (14)	0.74338 (8)	0.0207 (4)	
H2	0.678688	0.760457	0.726235	0.025*	
O3	−0.1792 (3)	0.92508 (10)	0.60406 (5)	0.0200 (3)	
C3	0.5636 (4)	0.78553 (14)	0.78973 (8)	0.0238 (5)	
H3	0.696009	0.754944	0.803896	0.029*	
C4	0.3851 (5)	0.82577 (14)	0.81637 (7)	0.0256 (5)	
H4	0.393972	0.823083	0.848482	0.031*	
O4	−0.5423 (3)	0.99473 (11)	0.61129 (5)	0.0260 (4)	
O5	1.3524 (3)	0.50427 (9)	0.51440 (5)	0.0180 (3)	
C5	0.1956 (5)	0.86955 (14)	0.79529 (7)	0.0229 (5)	
H5	0.070134	0.896356	0.813204	0.027*	
C6	0.1819 (4)	0.87587 (13)	0.74778 (7)	0.0173 (4)	
O6	1.2451 (3)	0.65830 (10)	0.47779 (5)	0.0213 (3)	
H6	1.255 (6)	0.6333 (13)	0.4577 (9)	0.032*	
H10	1.268 (5)	0.7058 (17)	0.4757 (9)	0.021 (7)*	
C7	−0.0186 (4)	0.92620 (12)	0.72964 (7)	0.0169 (4)	
H7	−0.137447	0.948897	0.750431	0.02*	
O7	0.8331 (3)	0.67403 (9)	0.54208 (5)	0.0169 (3)	
O8	0.4592 (3)	0.67902 (10)	0.57700 (5)	0.0233 (4)	
C8	−0.2649 (4)	0.99514 (13)	0.67389 (7)	0.0147 (4)	
H8	−0.40969	0.983539	0.694712	0.018*	
C9	−0.2066 (4)	1.09107 (13)	0.67467 (7)	0.0175 (4)	
H9	−0.369691	1.120986	0.668994	0.021*	
O9	0.7962 (3)	0.54667 (10)	0.47266 (5)	0.0199 (3)	
H11	0.639 (4)	0.5298 (17)	0.4845 (9)	0.036 (8)*	
C10	−0.1127 (5)	1.11970 (13)	0.72084 (7)	0.0257 (5)	
H10A	0.052137	1.094098	0.726988	0.039*	
H10B	−0.233321	1.102247	0.744205	0.039*	
H10C	−0.09647	1.181214	0.721102	0.039*	
C11	−0.0264 (5)	1.11791 (15)	0.63772 (8)	0.0302 (5)	
H11A	−0.095199	1.101598	0.608178	0.045*	
H11B	0.137196	1.090352	0.642289	0.045*	
H11C	−0.004356	1.179195	0.638722	0.045*	
C12	−0.3387 (4)	0.96951 (13)	0.62616 (7)	0.0177 (4)	
H12	0.888 (6)	0.5219 (18)	0.4458 (9)	0.083 (13)*	
C13	1.3597 (4)	0.42803 (12)	0.53182 (7)	0.0152 (4)	
C14	1.5490 (4)	0.37100 (13)	0.51776 (8)	0.0218 (5)	
H14	1.667897	0.388197	0.495506	0.026*	
C15	1.5648 (4)	0.29095 (14)	0.53564 (8)	0.0225 (5)	
H15	1.694644	0.254278	0.525437	0.027*	
C16	1.3961 (5)	0.26255 (13)	0.56815 (7)	0.0234 (4)	
H16	1.40961	0.207391	0.580411	0.028*	
C17	1.2097 (5)	0.31627 (13)	0.58204 (7)	0.0213 (5)	
H17	1.090484	0.29721	0.603799	0.026*	
C18	1.1888 (4)	0.39903 (12)	0.56511 (7)	0.0154 (4)	
C19	0.9900 (4)	0.44974 (13)	0.58356 (7)	0.0169 (4)	
H19	0.878687	0.423325	0.604428	0.02*	
C20	0.7350 (4)	0.56826 (13)	0.59688 (7)	0.0150 (4)	
H20	0.587525	0.528907	0.596481	0.018*	
C21	0.7975 (4)	0.59083 (14)	0.64687 (7)	0.0186 (4)	
H21	0.913474	0.546104	0.658543	0.022*	
C22	0.5643 (5)	0.58942 (17)	0.67622 (8)	0.0289 (5)	
H22A	0.446763	0.633181	0.666033	0.043*	
H22B	0.611852	0.600033	0.707791	0.043*	
H22C	0.482905	0.534139	0.6739	0.043*	
C23	0.9366 (5)	0.67446 (15)	0.65091 (8)	0.0248 (5)	
H23A	1.075345	0.676035	0.62896	0.037*	
H23B	1.004679	0.680335	0.681614	0.037*	
H23C	0.819359	0.720835	0.644748	0.037*	
C24	0.6677 (4)	0.64671 (13)	0.56966 (7)	0.0150 (4)	

### Atomic displacement parameters (Å^2^)

**Table:**

	*U*^11^	*U*^22^	*U*^33^	*U*^12^	*U*^13^	*U*^23^
Cu1	0.01555 (12)	0.01609 (11)	0.01265 (11)	0.00322 (11)	−0.00113 (10)	−0.00101 (8)
O1	0.0201 (8)	0.0222 (7)	0.0153 (7)	0.0047 (7)	−0.0001 (6)	0.0007 (5)
N1	0.0144 (8)	0.0138 (8)	0.0149 (8)	0.0019 (7)	−0.0025 (6)	−0.0010 (6)
C1	0.0151 (10)	0.0138 (9)	0.0194 (9)	−0.0022 (8)	−0.0024 (8)	0.0005 (7)
O2	0.0259 (8)	0.0170 (7)	0.0160 (7)	0.0069 (7)	0.0024 (6)	0.0013 (6)
Cu2	0.01489 (11)	0.01297 (11)	0.01415 (11)	0.00229 (10)	0.00415 (10)	0.00287 (8)
N2	0.0142 (8)	0.0140 (8)	0.0145 (8)	0.0030 (7)	0.0020 (6)	0.0010 (6)
C2	0.0178 (11)	0.0195 (10)	0.0247 (11)	0.0016 (9)	−0.0010 (8)	0.0022 (8)
O3	0.0200 (7)	0.0238 (8)	0.0161 (7)	0.0059 (6)	−0.0036 (6)	−0.0054 (6)
C3	0.0235 (12)	0.0221 (10)	0.0257 (11)	0.0013 (10)	−0.0078 (9)	0.0050 (8)
C4	0.0328 (12)	0.0271 (11)	0.0168 (10)	0.0003 (12)	−0.0061 (10)	0.0025 (8)
O4	0.0216 (8)	0.0318 (9)	0.0246 (8)	0.0093 (7)	−0.0096 (6)	−0.0086 (7)
O5	0.0176 (8)	0.0147 (6)	0.0217 (7)	0.0011 (6)	0.0051 (6)	0.0040 (5)
C5	0.0298 (12)	0.0217 (11)	0.0172 (10)	0.0037 (10)	−0.0014 (9)	−0.0010 (8)
C6	0.0200 (10)	0.0164 (9)	0.0155 (9)	−0.0004 (9)	−0.0034 (8)	0.0000 (8)
O6	0.0331 (9)	0.0128 (7)	0.0181 (8)	−0.0045 (7)	0.0085 (7)	0.0004 (6)
C7	0.0194 (10)	0.0138 (9)	0.0174 (10)	0.0015 (8)	0.0005 (8)	−0.0022 (7)
O7	0.0207 (8)	0.0144 (6)	0.0157 (7)	0.0036 (6)	0.0034 (6)	0.0031 (5)
O8	0.0227 (8)	0.0216 (8)	0.0256 (8)	0.0093 (7)	0.0066 (6)	0.0032 (6)
C8	0.0139 (9)	0.0151 (9)	0.0152 (9)	0.0027 (8)	−0.0008 (7)	−0.0013 (7)
C9	0.0186 (9)	0.0136 (9)	0.0203 (10)	0.0018 (9)	−0.0011 (8)	−0.0001 (8)
O9	0.0178 (7)	0.0258 (8)	0.0163 (7)	−0.0040 (6)	0.0039 (6)	0.0011 (6)
C10	0.0297 (12)	0.0191 (10)	0.0282 (11)	−0.0009 (11)	−0.0040 (11)	−0.0054 (8)
C11	0.0385 (14)	0.0216 (11)	0.0305 (12)	−0.0026 (10)	0.0070 (11)	0.0028 (10)
C12	0.0177 (11)	0.0158 (9)	0.0197 (10)	0.0006 (8)	−0.0019 (8)	−0.0028 (7)
C13	0.0143 (9)	0.0146 (8)	0.0167 (9)	0.0005 (8)	−0.0019 (8)	−0.0019 (7)
C14	0.0182 (11)	0.0182 (10)	0.0289 (11)	0.0015 (8)	0.0049 (8)	−0.0014 (8)
C15	0.0194 (11)	0.0184 (10)	0.0298 (12)	0.0056 (9)	0.0001 (9)	−0.0041 (8)
C16	0.0309 (11)	0.0143 (9)	0.0249 (10)	0.0044 (11)	−0.0035 (11)	0.0004 (8)
C17	0.0270 (11)	0.0167 (10)	0.0203 (11)	0.0026 (9)	0.0041 (9)	0.0031 (8)
C18	0.0178 (9)	0.0139 (9)	0.0146 (9)	0.0020 (8)	−0.0001 (7)	0.0008 (7)
C19	0.0201 (10)	0.0168 (10)	0.0139 (9)	−0.0003 (8)	0.0038 (8)	0.0030 (7)
C20	0.0144 (9)	0.0157 (9)	0.0150 (9)	0.0024 (8)	0.0040 (7)	0.0007 (7)
C21	0.0197 (10)	0.0212 (10)	0.0149 (9)	0.0053 (9)	0.0025 (8)	0.0010 (8)
C22	0.0291 (13)	0.0383 (13)	0.0192 (10)	−0.0018 (12)	0.0094 (9)	−0.0006 (9)
C23	0.0247 (12)	0.0300 (11)	0.0198 (10)	−0.0033 (10)	−0.0002 (9)	−0.0029 (9)
C24	0.0161 (10)	0.0151 (9)	0.0137 (9)	0.0018 (8)	−0.0006 (7)	−0.0018 (7)

### Geometric parameters (Å, º)

**Table:**

Cu1—O1	1.8955 (15)	O5—C13	1.315 (2)
Cu1—N1	1.9205 (16)	C5—C6	1.408 (3)
Cu1—O2	1.9339 (16)	C6—C7	1.433 (3)
Cu1—O3	1.9629 (15)	O7—C24	1.272 (2)
O1—C1	1.299 (2)	O8—C24	1.237 (3)
N1—C7	1.284 (3)	C8—C12	1.518 (3)
N1—C8	1.462 (3)	C8—C9	1.554 (3)
C1—C2	1.415 (3)	C9—C11	1.511 (3)
C1—C6	1.417 (3)	C9—C10	1.520 (3)
Cu2—N2	1.9243 (17)	C13—C18	1.413 (3)
Cu2—O6	1.9411 (15)	C13—C14	1.413 (3)
Cu2—O5	1.9432 (14)	C14—C15	1.378 (3)
Cu2—O7	1.9956 (14)	C15—C16	1.387 (3)
Cu2—O9	2.3663 (16)	C16—C17	1.367 (3)
N2—C19	1.278 (3)	C17—C18	1.410 (3)
N2—C20	1.464 (3)	C18—C19	1.433 (3)
C2—C3	1.370 (3)	C20—C24	1.524 (3)
O3—C12	1.279 (3)	C20—C21	1.554 (3)
C3—C4	1.386 (3)	C21—C22	1.509 (3)
C4—C5	1.370 (3)	C21—C23	1.523 (3)
O4—C12	1.231 (3)		
			
O1—Cu1—N1	94.40 (7)	C5—C6—C7	116.7 (2)
O1—Cu1—O2	94.04 (7)	C1—C6—C7	123.11 (18)
N1—Cu1—O2	170.16 (7)	N1—C7—C6	125.1 (2)
O1—Cu1—O3	171.32 (7)	C24—O7—Cu2	114.52 (12)
N1—Cu1—O3	82.90 (7)	N1—C8—C12	107.65 (16)
O2—Cu1—O3	88.01 (7)	N1—C8—C9	113.31 (17)
C1—O1—Cu1	126.10 (14)	C12—C8—C9	109.13 (17)
C7—N1—C8	118.99 (17)	C11—C9—C10	110.89 (19)
C7—N1—Cu1	124.59 (15)	C11—C9—C8	113.03 (18)
C8—N1—Cu1	114.74 (12)	C10—C9—C8	111.79 (17)
O1—C1—C2	119.00 (19)	O4—C12—O3	125.1 (2)
O1—C1—C6	124.71 (19)	O4—C12—C8	118.02 (18)
C2—C1—C6	116.29 (19)	O3—C12—C8	116.81 (18)
N2—Cu2—O6	177.73 (7)	O5—C13—C18	123.56 (18)
N2—Cu2—O5	92.86 (6)	O5—C13—C14	119.67 (19)
O6—Cu2—O5	89.41 (7)	C18—C13—C14	116.76 (18)
N2—Cu2—O7	82.74 (6)	C15—C14—C13	121.4 (2)
O6—Cu2—O7	95.00 (7)	C14—C15—C16	121.7 (2)
O5—Cu2—O7	175.60 (6)	C17—C16—C15	118.1 (2)
N2—Cu2—O9	93.05 (7)	C16—C17—C18	122.1 (2)
O6—Cu2—O9	86.56 (7)	C17—C18—C13	119.99 (19)
O5—Cu2—O9	97.98 (6)	C17—C18—C19	116.49 (19)
O7—Cu2—O9	82.34 (6)	C13—C18—C19	123.52 (18)
C19—N2—C20	117.73 (17)	N2—C19—C18	125.9 (2)
C19—N2—Cu2	126.01 (15)	N2—C20—C24	107.80 (16)
C20—N2—Cu2	115.27 (12)	N2—C20—C21	111.48 (17)
C3—C2—C1	122.0 (2)	C24—C20—C21	111.23 (17)
C12—O3—Cu1	115.88 (13)	C22—C21—C23	111.35 (19)
C2—C3—C4	121.4 (2)	C22—C21—C20	111.56 (18)
C5—C4—C3	118.4 (2)	C23—C21—C20	112.23 (17)
C13—O5—Cu2	126.88 (13)	O8—C24—O7	125.85 (19)
C4—C5—C6	121.8 (2)	O8—C24—C20	117.06 (18)
C5—C6—C1	120.1 (2)	O7—C24—C20	117.07 (17)

### Hydrogen-bond geometry (Å, º)

**Table:**

*D*—H···*A*	*D*—H	H···*A*	*D*···*A*	*D*—H···*A*
O2—H2*A*···O9^i^	0.83	1.87	2.688 (2)	167
O2—H2*B*···O8	0.72 (3)	1.93 (3)	2.645 (2)	169 (4)
O6—H6···O3^ii^	0.72	2.07	2.784 (2)	171
O6—H10···O7^iii^	0.77 (3)	2.01 (3)	2.764 (2)	169 (3)
O9—H11···O5^iv^	0.94 (2)	1.80 (2)	2.738 (2)	172 (3)
O9—H12···O4^ii^	1.01 (2)	1.74 (2)	2.703 (2)	157 (3)

Symmetry codes: (i) *x*−1/2, −*y*+3/2, −*z*+1; (ii) *x*+3/2, −*y*+3/2, −*z*+1; (iii) *x*+1/2, −*y*+3/2, −*z*+1; (iv) *x*−1, *y*, *z*.
