# Supplementary figures and images for: Crystal structure and Hirshfeld surface analysis of mono/bis­(aqua-κO)[N-(2-oxido­benzyl­idene)valinato-κ3 O,N,O′]copper(II): dimeric Schiff base copper(II) complexes having different numbers of coordinated water mol­ecules
Source: Acta Crystallogr E Crystallogr Commun. 2023 Mar 21;79(Pt 4):361–6. doi: 10.1107/S2056989023002487 (PMC10088302; doi:10.1107/S2056989023002487)

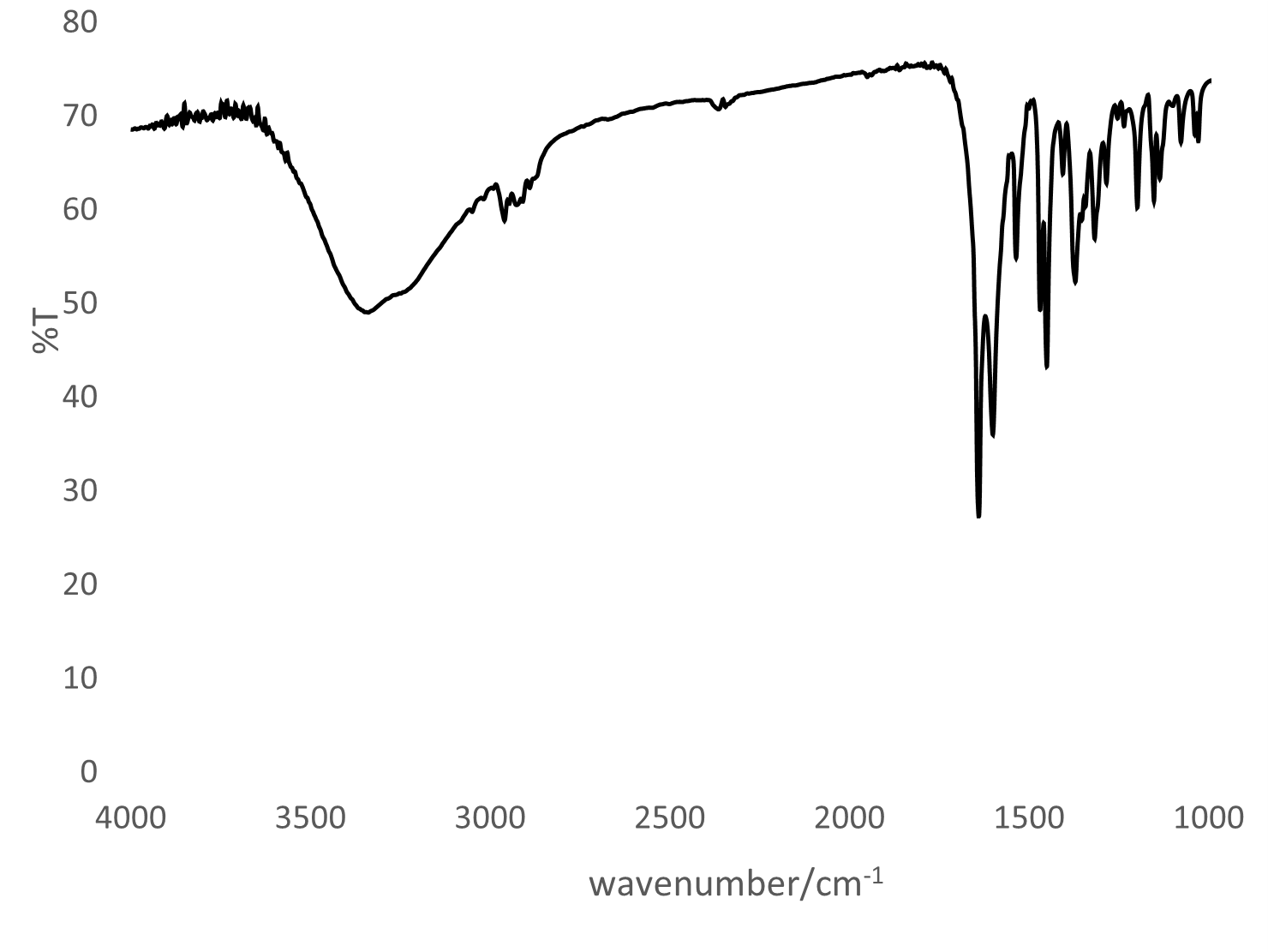

Supplement: Supplementary file 3 [file e-79-00361-sup3.tif]
